# *Nicotiana *species as surrogate host for studying the pathogenicity of *Acidovorax citrulli*, the causal agent of bacterial fruit blotch of cucurbits

**DOI:** 10.1111/mpp.12792

**Published:** 2019-04-01

**Authors:** Sy M. Traore, Noam Eckshtain‐Levi, Jiamin Miao, Anita Castro Sparks, Zhibo Wang, Kunru Wang, Qi Li, Saul Burdman, Ron Walcott, Gregory E. Welbaum, Bingyu Zhao

**Affiliations:** ^1^ School of Plant and Environmental Sciences Virginia Tech Blacksburg VA USA; ^2^ Department of Plant Pathology and Microbiology The Hebrew University of Jerusalem Rehovot Israel; ^3^ Department of Plant Pathology University of Georgia Athens GA USA

**Keywords:** *Acidovorax citrulli*, bacterial fruit blotch, effector‐triggered immunity, non‐host resistance, tobacco, type III effectors, watermelon

## Abstract

Bacterial fruit blotch (BFB) caused by *Acidovorax citrulli* is one of the most important bacterial diseases of cucurbits worldwide. However, the mechanisms associated with *A. citrulli* pathogenicity and genetics of host resistance have not been extensively investigated. We idenitfied *Nicotiana benthamiana* and *Nicotiana tabacum* as surrogate hosts for studying *A. citrulli* pathogenicity and non‐host resistance triggered by type III secreted (T3S) effectors. Two *A. citrulli* strains, M6 and AAC00‐1, that represent the two major groups amongst *A. citrulli *populations, induced disease symptoms on *N. benthamiana*, but triggered a hypersensitive response (HR) on *N. tabacum *plants. Transient expression of 19 T3S effectors from *A. citrulli* in *N. benthamiana* leaves revealed that three effectors, Aave_1548, Aave_2708, and Aave_2166, trigger water‐soaking‐like cell death in *N. benthamiana*. *Aave_1548* knockout mutants of M6 and AAC00‐1 displayed reduced virulence on *N. benthamiana* and melon (*Cucumis melo* L.). Transient expression of Aave_1548 and Aave_2166 effectors triggered a non‐host HR in *N. tabacum*, which was dependent on the functionality of the immune signalling component, *NtSGT1*. Hence, employing *Nicotiana* species as surrogate hosts for studying *A. citrulli* pathogenicity may help characterize the function of *A. citrulli* T3S effectors and facilitate the development of new strategies for BFB management.

## Introduction

Bacterial fruit blotch (BFB) of cucurbits is caused by the seed‐borne Gram‐negative bacterium *Acidovorax citrulli* (formerly *Acidovorax avenae* subsp. *citrulli*) (Schaad *et al.*, 2008). *A. citrulli* gained recognition after severe outbreaks occurred in watermelon fields in several USA states in the late 1980s. Subsequently, the pathogen has spread to many parts of the world, mainly by seed transmission, and has become a serious threat to the cucurbit industry worldwide (Burdman and Walcott, 2012). Several research groups have screened plant germplasm for resistance to BFB. While some cucurbit germplasm lines have been reported to be partially tolerant to *A. citrulli* (Bahar *et al.*, 2009a, Wechter *et al.*, 2011), to date, no cucurbit lines have been identified with complete resistance. Thus, all commercial cultivars of watermelon (*Citrullus lanatus* Thunb.) and melon (*Cucumis melo* L.) are susceptible to *A. citrulli*. Despite the economic importance of BFB, little is known about the molecular basis of *A. citrulli*‐cucurbit interactions (Bahar and Burdman, 2010; Burdman and Walcott, 2012).

Two major evolutionary lineages of *A. citrulli *have been identified through DNA fingerprinting and multi locus sequence typing analysis (Feng *et al.*, 2009; Walcott *et al.*, 2000). Group I strains have a broad host range that includes several cucurbit species but primarily threaten commercially produced melon (Burdman *et al.*, 2005; Walcott *et al.*, 2004). M6, an *A. citrulli *strain isolated from melon plants in Israel (Burdman *et al.*, 2005), is representative of Group I and has become the model Group I strain for pathogenicity studies. In contrast, Group II strains are highly virulent on watermelon but less virulent on melon and other cucurbit species (Eckshtain‐Levi *et al.*, 2014; Walcott *et al.*, 2004). The Group II representative strain, AAC00‐1, was isolated from watermelon in the USA and its genome has been completely sequenced (GenBank accession NC_008752).

Annotation of the AAC00‐1 genome revealed the presence of genes encoding components of a type III secretion system (T3SS). In many Gram‐negative plant pathogenic bacteria, the T3SS is responsible for secretion of effector proteins into the cytosol of their hosts (Hueck, 1998). Most type III secreted (T3S) effectors characterized so far contribute to suppression of host immunity in susceptible plants (Alfano and Collmer, 2004), while some are recognized by specific plant disease resistance (*R*) genes to induce effector‐triggered immunity (ETI) in resistant plants. Plant *R* genes mostly encode NB‐LRR type proteins and their functionality frequently requires the conserved immune signalling component, SGT1 (Peart *et al.*, 2002). Mutation of T3SS genes from both Groups I and II *A. citrulli* strains abolished their ability to trigger hypersensitive response (HR) in non‐host plants (i.e. tobacco and tomato) and pathogenicity in watermelon and melon plants (Bahar and Burdman, 2010; Johnson *et al.*, 2011; Ren *et al.*, 2009).

Analysis of the *A. citrulli* AAC00‐1 genome also revealed at least 11 putative T3S effector genes, based on their homology to known effectors in other bacterial species (Eckshtain‐Levi *et al.*, 2014). Comparative analyses of the 11 effector genes cloned from 22 *A. citrulli* strains indicated that Groups I and II strains of *A. citrulli* have evolved different T3S effector repertoires. Moreover, all assessed Group I strains were found to lack or have truncated *Aave_2166*, *Aave_2708*, and *Aave_3062* genes (gene names are according to the AAC00‐1 annotation) (Eckshtain‐Levi *et al.*, 2014). It was proposed that differences in the repertoire of T3S effectors between the groups contribute to observed differences in host preferential association between Groups I and II *A. citrulli *lineages. However, thus far, none of these putative *A. citrulli* effectors have been functionally characterized in regards to their contribution to virulence on susceptible host plants or their ability to trigger ETI on resistant plants.

Model plant species have been used for studying important plant diseases (Glazebrook *et al.*, 1997; Goodin *et al.*, 2008). For instance, the tomato pathogen, *Pseudomonas syringae* pv. *tomato* (*Pst*) strain DC3000, is pathogenic on certain genotypes of *Arabidopsis thaliana* (Dangl *et al.*, 1992; Dong *et al.*, 1991; Whalen *et al.*, 1991). Subsequently the molecular interactions between Arabidopsis and *Pst* DC3000 have been extensively studied, leading to a detailed understanding of the molecular mechanisms of bacterial virulence and plant immunity. Virus‐host interactions have been studied for several decades in tobacco species, including *Nicotiana benthamiana* and *Nicotiana tabacum*, largely because of their susceptibility to various viruses (Goodin *et al.*, 2008). Other plant pathogens, including bacteria (Metz *et al.*, 2005, Wei *et al.*, 2007), fungi (Dean *et al.*, 2005; Rivas‐San Vicente *et al.*, 2013), and oomycetes (Chaparro‐Garcia *et al.*, 2011), have also been studied on *N.* *benthamiana*. In addition, both *N. benthamiana* and *N. tabacum* can be easily transformed by *Agrobacterium* *tumefaciens*, allowing efficient protein expression through *Agrobacterium*‐mediated transient assays. Tobacco rattle virus (TRV)‐based virus‐induced gene silencing (VIGS) has been developed to study potential immunity genes in *N. benthamiana* (Zhu and Dinesh‐Kumar, 2008). Recently, the draft genomes of *N. benthamiana* and *N. tabacum* have been determined (Bombarely *et al.*, 2012). The aforementioned factors make *N. benthamiana* and *N. tabacum* attractive model plants for studying pathogen‐host interactions, and in particular *A. citrulli*‐plant interactions.

The goals of this study were: (i) to compare the disease development of *A. citrulli* on *N. benthamiana* and *N. tabacum* along with its natural host watermelon plants; (ii) to determine if *N. benthamiana *could serve as a surrogate host for studying pathogenicity mechanisms of *A. citrulli*; (iii) to characterize the virulence and avirulence functions of selected T3S effector genes of *A. citrulli* on *N. benthamiana *and *N. tabacum*. Here we report that *N. benthamiana* can function as a surrogate host for studying *A. citrulli* pathogenicity. One T3S effector, Aave_1548, was critical for *A. citrulli* viruence. Transient expression of three T3S effectors (Aave_1548, Aave_2708 and Aave_2166) triggered a non‐host HR in *N. tabacum *that was dependent on the function of NtSGT1. This is the first report on functional characterization of *A. citrulli* T3S effectors*. *Employing *Nicotiana* species as surrogate hosts for studying *A. citrulli* pathogenicity may advance the understanding of the function of *A. citrulli* T3S effectors and facilitate the development of new strategies for BFB management.

## Results

### *N. benthamiana* was susceptible to *A. citrulli *infection while *N. tabacum* was resistant

**Figure 1:**
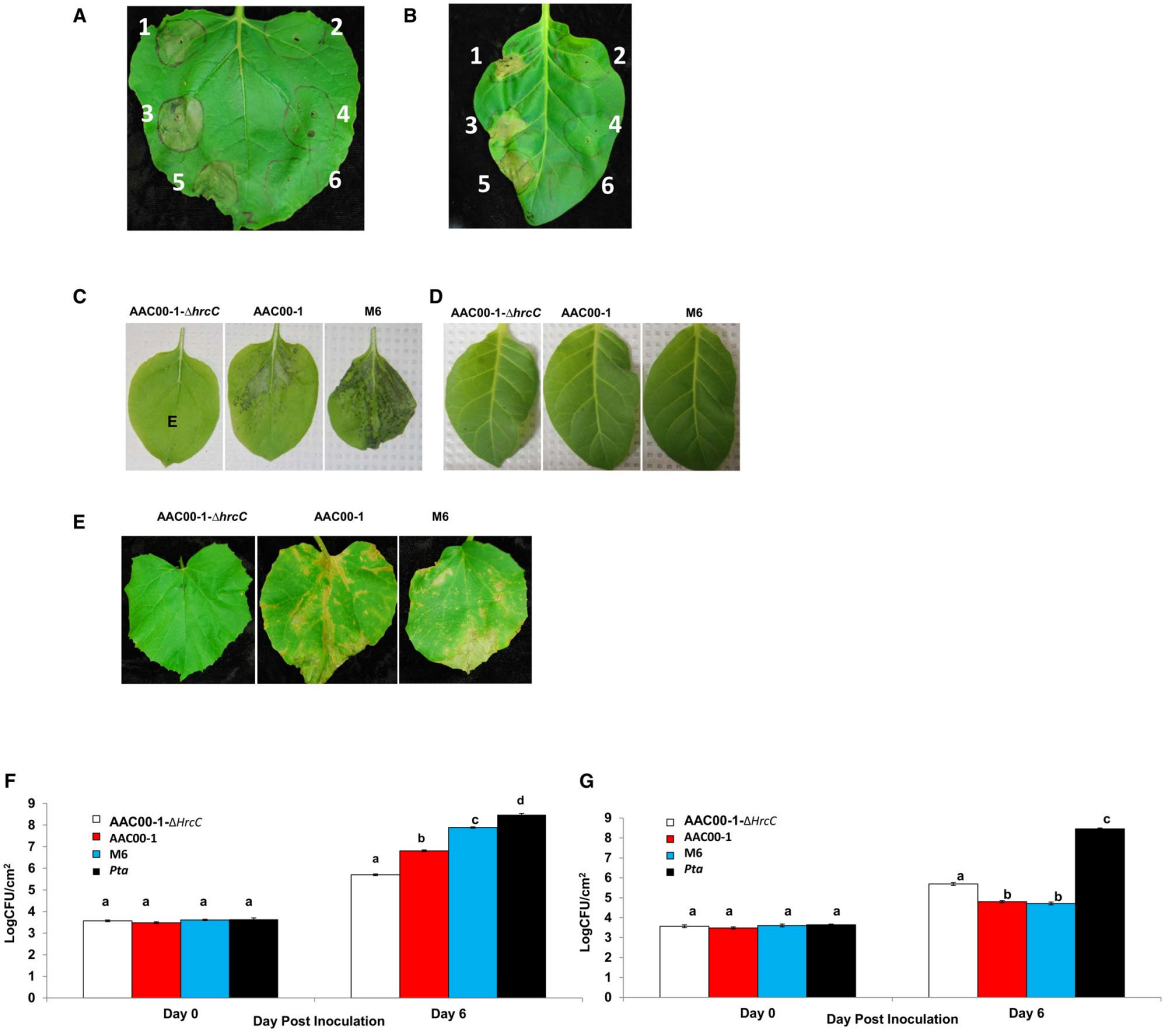
Inoculation of *Acidovorax citrulli *onto *Nicotiana benthamiana *and *Nicotiana tabacum *plants*. *(A, B) *A. citrulli* strains trigger a water‐soaking‐like cell death response in *N. benthamiana *(A) and a hypersensitive response (HR)‐like cell death response in *N. tabacum* (B) plants. Leaves of 4‐week‐old plant*s *were infiltrated with (1) AAC00‐1, (2) AAC00‐1‐Δ*hrcC*, (3) M6, (4) M6‐Δ*hrcV, *or (5) *Pseudomonas syringae *pv.* tabaci* (*Pta*) 11528, (6) *Pta*‐Δ*hrcV* at ~0.3 × 10^8 ^CFU/mL. The plants were incubated at 25 °C for 3 days before photographs were taken. (C, D, E) Symptoms on *N. benthamiana* (C), *N. tabacum* (D), and watermelon (E) plants after spray‐inoculation with different *A. citrulli* strains. Four‐week‐old plants were spray‐inoculated with AAC00‐1‐Δ*hrcC*, AAC00‐1 or M6 at ~0.2 × 10^8 ^CFU/mL. The inoculated plants were incubated at 30 °C for 2 days before photographs were taken. (F, G) Bacterial growth of *A. citrulli* strains on *N. benthamiana* (F) and *N. tabacum* (G) plants. Leaves of 4‐week‐old plants were infiltrated with AAC00‐1‐Δ*hrcC*, AAC00‐1, M6 and *Pta* 11528 at ~0.3 × 10^5^ CFU/mL. Bacterial populations were monitored at 0 days and 6 days post‐inoculation (dpi). Data represent means ± standard errors (SE) from one representative experiment of three with similar results, with three replicates per treatment. Different letters indicate significant differences (*P* ≤ 0.05) amongst treatments within each time point by Tukey–Kramer HSD test.

To determine if *A. citrulli* strains are pathogenic on *N. benthamiana*, we inoculated 4‐week‐old plants by tissue infiltration and spray‐inoculation methods. Infiltration of high concentrations of bacterial cell suspensions (~0.3 × 10^8 ^CFU/mL) into *N. benthamiana* leaves triggered a strong water‐soaking‐like cell death at the inoculation site by 2 days post‐inoculation (dpi) (Fig. 1A). When infiltrated with lower concentrations (~0.3 × 10^5 ^CFU/mL), the inoculated leaves of *N. benthamiana* turned chlorotic and a black necrosis developed along the veins at 6 dpi (data not shown). At 9 dpi, brown necrosis developed on the main stem near the petiole of inoculated leaves. Plating of petiole and stem extracts confirmed the presence of *A. citrulli *(data not shown). As a control, *N. benthamiana* plants were inoculated with a suspension of *Pseudomonas syringae *pv.* tabaci* (*Pta*) (Oh and Collmer, 2005) containing ~0.3 × 10^8^ CFU/mL. Inoculation with *Pta *triggered a similar water‐soaking‐like cell death (Fig. 1A) in *N. benthimiana*. Disease symptoms did not develop on plants inoculated with the M6 and AAC00‐1 mutants impaired in the T3SS genes *hrcV* (Bahar and Burdman, 2010) and *hrcC* (Johnson *et al.*, 2011), respectively (Fig. 1A).

To determine if *A. citrulli* could infect other tobacco species, we infiltrated high concentrations (~0.3 × 10^8 ^CFU/mL) of M6 and AAC00‐1 cell suspensions into *N. tabacum *leaves. Both M6 and AAC00‐1 triggered a rapid and strong HR‐like cell death 24 h after infiltration (Fig. 1B). In contrast, T3SS mutants M6‐Δ*hrcV* and AAC00‐1Δ*hrcC* failed to trigger HR (Fig. 1B). These results support that secretion of T3S effectors from *A. citrulli* trigger non‐host HR in *N. tabacum*.

We further characterized the susceptibility of *N. benthamiana* and the resistance of *N. tabacum* to *A. citrulli *by the spray‐inoculation method (Fig. 1C and D). Four‐week‐old plants of both species were spray‐inoculated with bacterial cell suspensions containing ~0.2 × 10^8 ^CFU/mL. Spray‐inoculation of *A. citrulli* strains M6 and AAC00‐1 onto *N. benthamiana* led to the development of necrotic lesions similar to those observed in the natural host, watermelon (Fig. 1C and E). Interestingly, inoculation with the Group I strain M6 consistently resulted in greater disease severity than the Group II strain, AAC00‐1. This observation is important considering that Group II strains are highly virulent on watermelon but weakly to moderately virulent on other cucurbits, while Group I strains are moderately to highly virulent in diverse cucurbits. Differences in disease severity between these two strains also correlated with differences in bacterial populations at 6 dpi (Fig. 1F): at 6 dpi, M6 populations were significantly (*P* ≤ 0.05) higher than those of AAC00‐1 in *N. benthimiana*. AAC00‐1Δ*hrcC* did not induce disease symptoms on *N. benthamiana* plants (Fig. 1C) and its growth was significantly (*P* ≤ 0.05) impaired in *N. benthamiana* leaves relative to the wild‐type strains (Fig. 1F).

In contrast to *N. benthamiana*, symptoms were not observed on *N. tabacum* plants spray‐inoculated with different *A. citrulli* strains (Fig. 1D). These results were in agreement with the observed HR induced by *A. citrulli* wild‐type strains in *N. benthamiana.* Also in agreement with these findings, in infiltration assays, populations of AAC00‐1 and M6 were significantly (*P* ≤ 0.05) lower than those of the pathogenic control *Pta* at 6 dpi. Moreover, at this time, populations of these strains were about 2 to 3 orders of magnitude lower than those observed for the same strains in *N. benthamiana* (Fig. 1F and G). Overall, virulence assays indicate that *N. benthamiana* is susceptible to infection by *A. citrulli*, while *N. tabacum* is resistant to this pathogen. Additionally, these responses are T3SS‐dependent.

### Transient expression of four T3S effectors induced water‐soaking in *N. benthamiana* and cell death in *N. tabacum*

**Figure 2:**
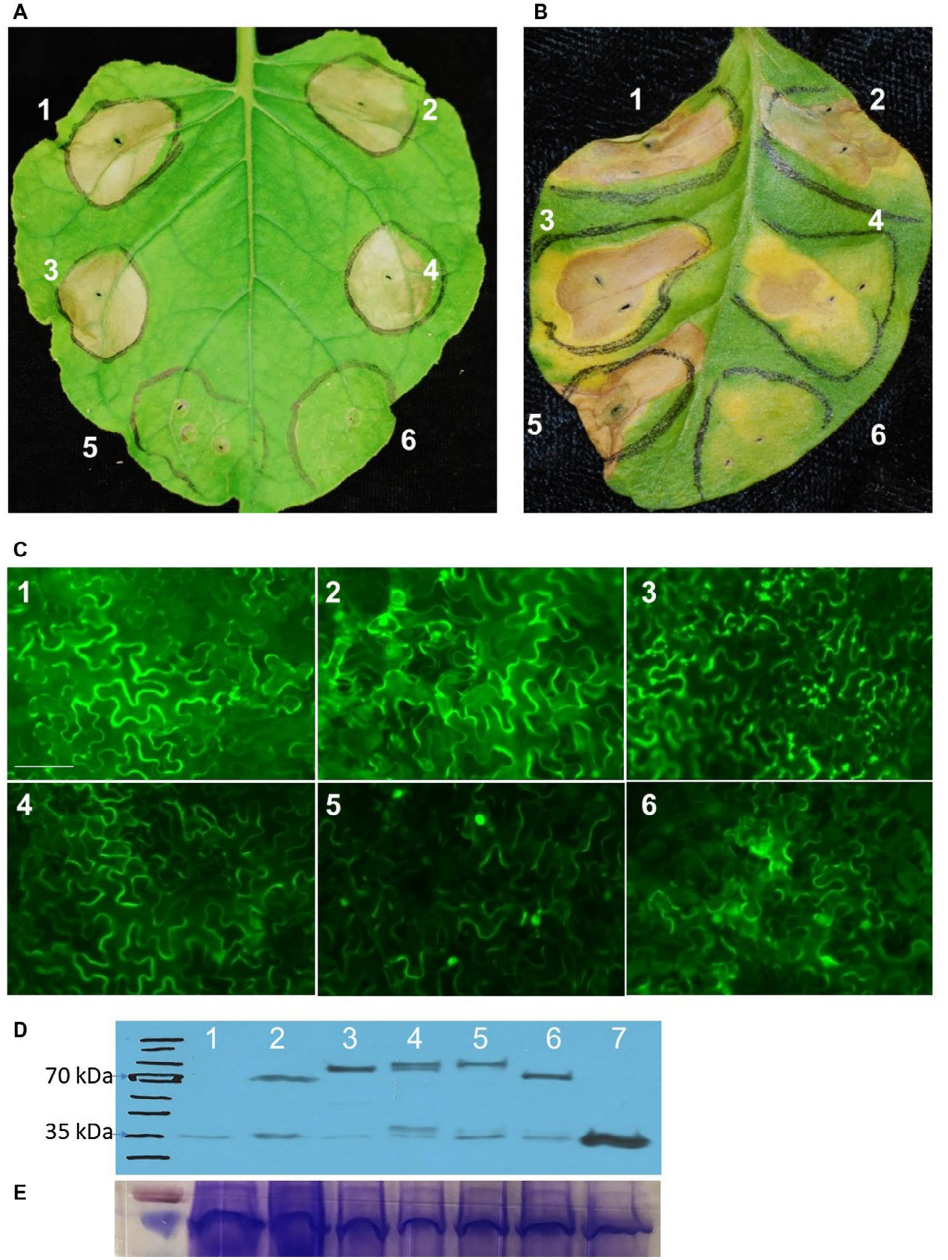
Transient expression of three *Acidovorax citrulli* T3S effectors trigger water‐soaking‐like cell death on *Nicotiana benthamiana *and hypersensitive response (HR)‐like cell death on *Nicotiana tabacum *plants*. *(A) *Agrobacterium‐*mediated transient expression of T3S effectors in *N. benthamiana*. Effectors Aave_1548 from strains M6 and AAC00‐1, and Aave_2166 and Aave_2708 but not Aave_3062 from strain AAC00‐1 triggered water‐soaking like cell death on *N. benthamiana* at a concentration of ~0.3 × 10^8^ CFU/mL. Pictures were taken at 2 dpi. (B) Transient expression of Aave_2166 and Aave_1548 but not Aave_3062 triggered HR‐like cell death on *N. tabacum* at 1 dpi. (C) Yellow florescent protein (YFP) fluorescence showing expression of effectors in *N. benthamiana* cells. The fluorescent signal of effector**‐**YFP fusion proteins expressed in *N. benthamiana* was detected by fluorescence micorscopy. Negative control was an *Agrobacterium* strain carrying an empty vector. Bars represent 20 µm. (D). Effector‐YFP‐HA fusion proteins were detected by Western blot. (1) Non–transanformed control plant (2) pEG101‐Aave_2166, (3) pEG101‐Aave_2708, (4) pEG101‐Aave_1548‐AAC00‐1, (5) pEG101‐Aave_1548‐M6, (6) pEG101‐Aave_3602, (7) pEG101‐YFP‐HA. € The same set of protein samples were also loaded in PAGE gel, and stained with Coomassie Blue dye to confirm the equal loadings.

In total, 19 putative T3S effector genes were cloned, 11 from AAC00‐1 and eight from M6*. *Only eight effector genes were cloned from M6 because three, *Aave_2166*, *Aave_2708* and *Aave_3062 *are either absent or truncated in this strain (Eckshtain‐Levi *et al.*, 2014). To determine if any of the 19 T3S effector genes contribute to the ability of *A. citrulli *to trigger water‐soaking‐like cell death in *N. benthamiana* and HR in *N. tabacum*, each effector gene was sub‐cloned into the binary vector pEG101‐SacB/R for *Agrobacterium*‐mediated transient assays (Traore and Zhao, 2011). The pEG101‐SacB/R vector has a yellow florescent protein (YFP) tag fused to the C‐terminus of the expression cassette, which allows monitoring of effector expression *in planta* by fluorescence microscopy. Transient expression of three T3S effector genes from AAC00‐1 (*Aave_1548, Aave_2166* and *Aave_2708*), and one T3S effector gene from M6 (*Aave_1548*), triggered water‐soaking‐like cell death in *N. benthamiana *(Fig. 2A) and HR‐like cell death in *N. tabacum *(Fig. 2B). The water‐soaking‐like, cell death phenotype on *N. benthamiana* was observed 48 h after inoculation, while the HR‐like cell death on *N. tabacum *plants was observed within 24 h after inoculation. Of the three genes cloned from AAC00‐1, *Aave_2166* triggered the strongest cell death on *N. tabacum*. Interestingly, *Aave_1548 *from M6 trigged stronger cell death compared to *Aave_1548 *from AAC00‐1 (Fig. 2B)*. *We have previously shown that the nucleotide sequence of the central region of *Aave_1548* is highly variable between Groups I and II A. *citrulli *strains (Eckshtain‐Levi *et al.*, 2014) (shown for AAC00‐1 and M6 in Supplementary Fig. S2). The expression of all effector‐YFP fusion proteins was confirmed by flourescence microscopy (Fig. 2C) and Western blot analysis (Fig. 2D and E).

### *Aave_1548* and *Aave_2166* are important for *A. citrulli* virulence on *N. benthamiana *plants

**Figure 3:**
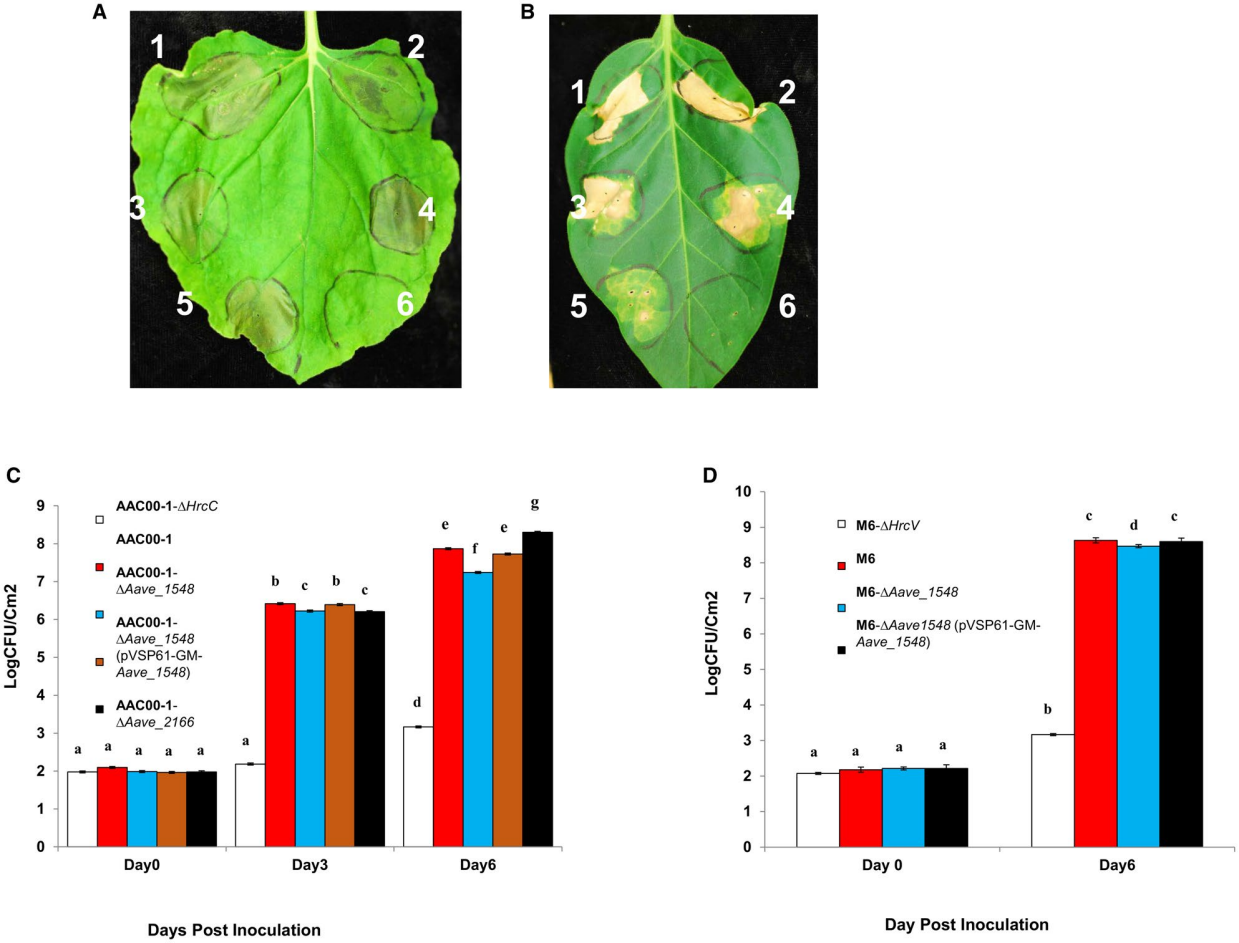
Effects of impairment of T3S effector genes in *Acidovorax citrulli* on symptom development and bacterial population growth on *Nicotiana benthamiana* and *Nicotiana tabacum* plants. (A, B) Inoculation of *N. benthamiana* (A) or *N. tabacum *(B) leaves with *A. citrulli* strains: 4‐week‐old *N. benthamiana *leaves were infiltrated with strains (1) AAC00‐1, (2) AAC00‐1‐Δ*Aave_2166, *(3) AAC00‐1‐Δ*Aave_1548, *(4) M6, (7) M6‐Δ*Aave_1548 *at a concentration of ~0.3 × 10^8^ CFU/mL, (6) 10 mM MgCl_2_. Mutant strains AAC00‐1‐Δ*Aave_2166, *AAC00‐1*‐*Δ*Aave_1548 *and M6‐Δ*Aave*_*1548*, triggered weaker cell death phenotypes compared to the wild‐type strains on *N. benthamiana.* Both wild‐type and mutant *A. citrulli* strains triggered strong hypersensitive response (HR) on *N. tabacum*. Pictures were taken at 2 dpi for *N. benthamiana *and 4 dpi for *N. tabacum *plants. (C, D) *In* *planta *population growth of *A. citrulli* strains on *N. benthamiana *plants: 4‐week‐old *N. benthamiana *leaves were spray‐inoculated with *A. citrulli* strains at concentrations of ~0.2 × 10^8^ CFU/mL. Bacterial population growth was monitored at 0, 3 and 6 dpi for AAC00‐1 and AAC00‐1‐derived strains (C) and 0 and 6 dpi for M6 and M6‐derived strains (D). AAC00‐1‐Δ*Aave_1548*(pVSP61‐GM‐Aave_1548) is a plasmid‐borne complemented strain of the *Aave_1548 *deletion mutant. Data represents means ± standard errors (SE) of one representative experiment of three with similar results, with there replicates per treatment. Different letters indicate significant differences (*P* ≤ 0.05) amongst treatments within each time point by Tukey–Kramer HSD test.

To determine if any T3S effector genes contribute to *A. citrulli* virulence on *N. benthamiana*, *Aave_1548* and *Aave_2166* were mutated in AAC00‐1 and M6 by marker‐exchange mutagenesis. *N. benthamiana* plants were inoculated with *Aave_1548 *mutants and complemented strains using tissue infiltration for phenotype observation and spray‐inoculation for population growth assessment. When inoculated at a high concentration (~0.3 × 10^8 ^CFU/mL), the *Aave_1548 *mutant strains triggered weaker cell death in *N. benthamiana* than the wild type and complemented strains (Fig. 3A). Similarly, at 6 dpi, AAC00‐1‐Δ*Aave_1548 *grew significantly (*P* ≤ 0.05) less than both the wild type and the *Aave_1548* complemented strain on *N. tabacum *(Fig. 3B). M6Δ‐*Aave_1548* mutant also grew to significantly (*P* ≤ 0.05) lower populations than the wild‐type strain in *N. benthamiana* (Fig. 3C). These results suggest that effector Aave_1548 significantly contributes to the virulence of both M6 and AAC00‐1 on *N. benthamiana* (Fig. 3A–C).

The AAC00‐1Δ‐*Aave_2166 *mutant triggered a weaker cell death response than the wild‐type strain when infiltrated at high concentrations into *N. benthamiana* leaves (Fig. 3A). At 3 dpi, the *Aave_2166* mutant grew to significantly (*P* ≤ 0.05) lower populations than wild‐type AAC00‐1 (Fig. 3B). However, at 6 dpi, AAC00‐1‐Δ*Aave_2166* grew to a significantly (*P* ≤ 0.05) higher population than the wild‐type strain on *N. benthamiana* (Fig. 3B). We were unable to generate a complemented strain for this mutant. Attempts to amplify and clone a DNA fragment containing the full length of this gene were unsuccessful, probably because of its high G + C content.

When inoculated at high concentrations (~0.3 × 10^8 ^CFU/mL) into *N. tabacum*, the *Aave_1548* and *Aave_2166* deletion mutants still triggered HR‐like cell death (Fig. 3D), which suggests that there are multiple T3S effectors or other components of *A. citrulli* that trigger HR on *N. tabacum *in a T3SS‐dependent manner. These results suggest that two T3S effector genes, *Aave_1548* and *Aave_2166*, contribute substantially to *A. citrulli* virulence and its ability to infect *N. benthamiana* plants.

### *Aave_1548* contributes to *A. citrulli* virulence on melon plants

To further characterize the roles of effectors Aave_1548 and Aave_2166 in *A. citrulli* virulence, we compared both the wild‐type and mutant strains on melon plants by seedling transmission assays. M6‐*ΔAave_1548* and AAC00‐1‐*ΔAave_1548* showed significant (*P* ≤ 0.05) reductions in BFB seed transmission efficiency compared to their corresponding wild‐type strains (Table 1). Melon seeds inoculated with AAC00‐1‐*ΔAave_2166* developed seedlings that weighed not significantly different from those inoculated with wild‐type AAC00‐1 (Table 1). Results from seed transmission assays confirmed that *Aave_1548*, which is present in all *A. citrulli *strains tested so far (Eckshtain‐Levi *et al.*, 2014), is important for virulence on both surrogate and natural host plants. While *Ave_2166 *may have either no virulence function or with redudannt functions with other effectors on host plants.

**Table 1:** Seed transmission assays to compare the virulence of wild type and mutant strains of f *Aciovorax* *citrulli* AAC00‐1 and M6.*

Bacterial strains	Plant weight (mg)†	Disease severity‡
AAC00‐1	187 ± 43*	3.8 ± 0.5
AAC00‐1‐Δ*Aave_2166*	208 ± 38*	3.1 ± 0.4
AAC00‐1‐Δ*Aave_1548*	417 ± 32†	0.6 ± 0.2
AAC00‐1‐Δ*Aave_1548 *(pVSP61‐GM‐Aave_1548)	190 ± 47*	3.6 ± 0.4
Control (H_2_O)	460 ± 61†	0
M6	493.77 ± 75.39*	5.2 ± 0.2
M6‐Δ*Aave_1548*	899.83 ± 110†	3. 8 ± 0.3
Control (H_2_O)	1993.88 ± 93‡	0

* Hybrid cantaloupe/muskmelon seeds (cv. Athena) were used for inoculations with AAC00‐1 and AAC00‐1 mutants, while melon cv. Ofir seeds were used for inoculations of M6 and M6 mutants. All inoculated and control seeds were incubated in bacterial cell suspensions (~1 × 10^6 ^and 1 × 10^7^ CFU/mL for Athena and Ofir seeds, respectively). Control seeds were subjected to the same treatment but with water) for 2 h. Infected seeds were blot dried and planted, four seeds/per pot with two pots for each treatment. The weights of the seedlings were measured at 12 days post‐inoculation and compared to the control (H_2_O).

† In plant weight values, different letters indicate significant differences between treatments (*P* ≤ 0.05) by Tukey–Kramer HSD test.

‡ Disease severity was scored using a scale of 0 to 7, based on plant weight values of inoculated plants relative to the average shoot weight of non‐inoculated controls (Bahar *et al.*, 2009b): 0, weight higher than 90% of average control weight; 1 to 5, weight equal to 76%–90%, 61%–75%, 46%–60%, 31%–45% and 16%–30% of average control weight, respectively; 6, weight equal to or lower than 15% of average control weight; 7, dead seedling. Data was presented as mean ± standard error, *n* = 8.

### Silencing the *NtSGT1 *gene in *N. tabacum* compromised its resistance to *A. citrulli*

**Figure 4:**
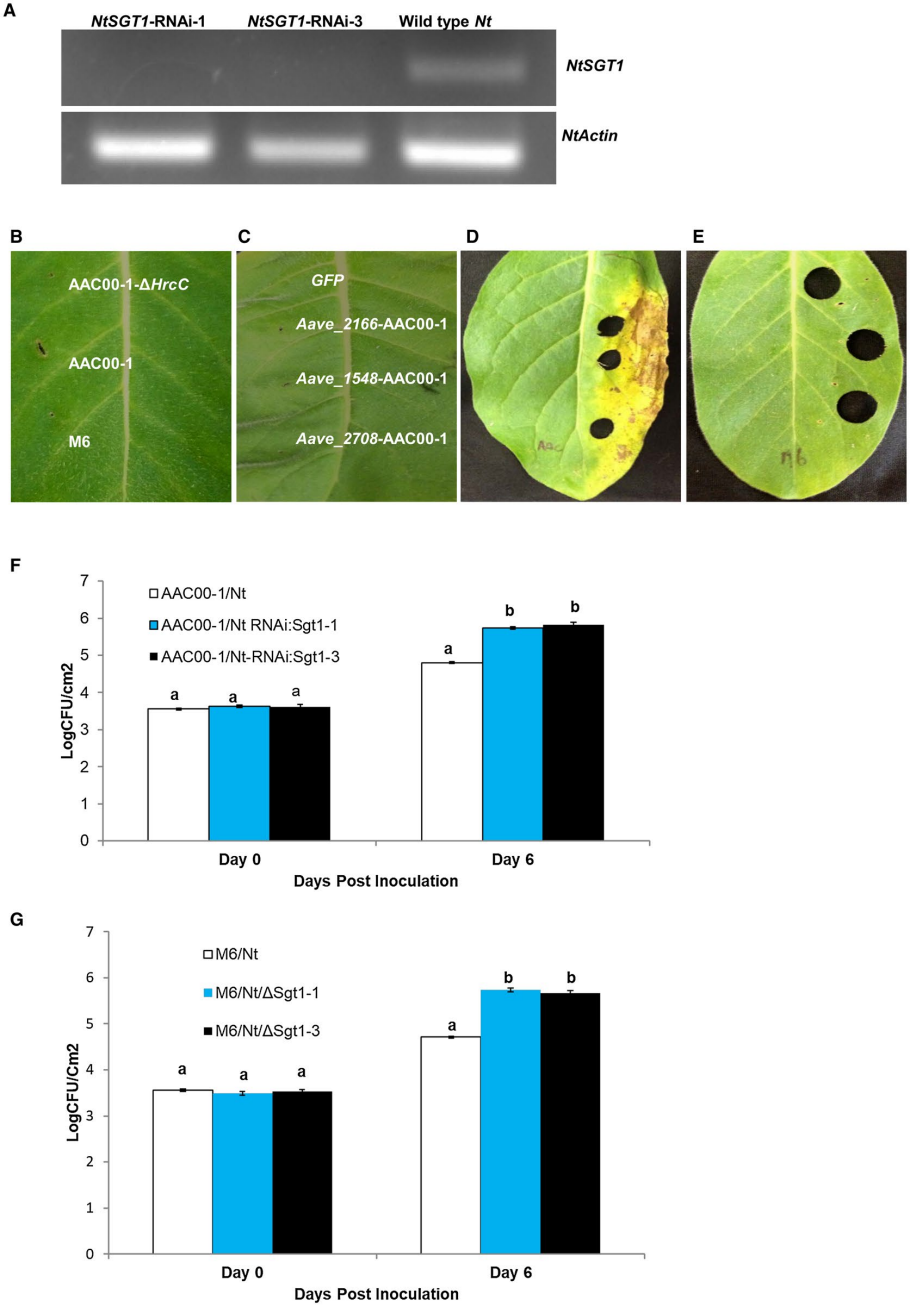
Silencing of *NtSGT1* in *Nicotiana tabacum* suppressed the hypersensitive response (HR) phenotype triggered by *Acidovorax citrulli* strains and individual T3S effectors. (A) Validation of *NtSGT1* gene silencing by Reverse Transcriptase‐Polymerase Chain Reaction (RT‐PCR) analysis. *NtSGT1* gene specific primers flanking the region that is not part of DNA fragment used for the RNAi construct was employed for RT‐PCR analysis. *NtActin* primers were used as internal control. cDNAs isolated from two independent *NtSGT1*‐RNAi lines (*NtSGT1‐RNAi*‐1 and *NtSGT1‐RNAi*‐3) and the wild‐type *N. tabacum* (*Nt*) plants were used for RT‐PCR. (B) HR was not triggered in *NtSGT1*‐RNAi lines inoculated with *A. citrulli* strains. Two independent *NtSGT1*‐RNAi lines were inoculated with AAC00‐1, M6 and AAC00‐1‐*∆hrc*C strains at ~0.3 × 10^8^ CFU/mL (shown for one of the lines). Pictures were taken at 2 dpi. (C) *Agrobacterium*‐mediated transient expression of T3S effectors did not trigger HR in *NtSGT1*‐RNAi plants. *Agrobacterium tumefaciens *strains carrying the effector genes *Aave_2166*, *Aave_1548* and *Aave_2708* from strain AAC00‐1 or a *GFP* gene were inoculated on *NtSGT1*‐RNAi plants at the concentration of ~0.3 × 10^8^ CFU/mL. Pictures were taken at 2 dpi. (D, E) *A. citrulli* strain AAC00‐1 triggered disease lesions on *NtSGT1*‐RNAi plants (D) while M6 did not (E). The *NtSGT1*‐RNAi transgenic plants were infiltrated with *A. citrulli* strains at a concentration of ~0.3 × 10^5^ CFU/mL. Pictures were taken at 9 dpi. (F, G) In *planta* growth of *A. citrulli* populations on *NtSGT1*‐RNAi and wild‐type *N. tabacum* plants. AAC00‐1(F) and M6 (G) were infiltrated‐inoculated onto *NtSGT1*‐RNAi plants at the concentration of ~0.3 × 10^5^ CFU/mL. Data represent means ± standard errors (SE) of one representative experiment of three with similar results, with three replicates per treatment. Different letters indicate significant differences between treatments (*P* ≤ 0.05) within each time point by Tukey–Kramer HSD test.

At least three *A. citrulli* T3S effectors elicited HR in *N. tabacum*, but T3SS mutants of *A. citrulli* failed to induce HR. Therefore, we hypothesized that this HR is the result of specific recognition of T3S effector(s) by unknown cognate plant *R* gene(s). The function of many *R* genes depends on the presence of the conserved immune signalling component, *SGT1 *(Peart *et al.*, 2002). It was also reported that pepper SGT1 interacts with the *Xanthomonas* effector AvrBsT, a homologue of Aave_2166, that is required for AvrBsT‐triggered cell death in pepper (Kim *et al.*, 2014). Therefore, we attempted to silence *NtSGT1* using RNAi to determine if it is required to elicit HR in *N.* *tabacum* in response to *A. citrulli*. Two independent *NtSGT1*‐RNAi transgenic lines were used for this study, and *NtSGT1* silencing was confirmed by Reverse Transcription‐Polymerase Chain Reaction (RT‐PCR) (Fig. 4A). Transgenic and non‐transgenic *N. tabacum* plants were used for inoculation and *in planta* bacterial growth assays with *A. citrulli* strains. Inoculation with AAC00‐1 and M6 failed to trigger HR cell death in the RNAi*‐NtSGT1* transgenic *N. tabacum* plants (Figs 1B and 4B). Compared to the phenotypes observed with non‐transgenic *N. tabacum* plants, transient expression of *Aave_2166 *and *Aave_1548 *from AAC00‐1, as well as *Aave_1548* from M6 failed to trigger the cell death phenotype on the RNAi‐*NtSGT1* plants (Figs 2B and 4C), even though the fluorescent signal of the effector‐YFP fusion proteins was detected (data not shown).

To further test whether silencing *NtSGT1* in *N. tabacum* could suppress its resistance to *A. citrulli*, the population dynamics of AAC00‐1 and M6 on RNAi‐*NtSGT1* plants were monitored. AAC00‐1 induced weak lesions on inoculated leaves at 9 dpi, while M6 failed to induce lesions by 9 dpi (Fig. 4D and E). Despite this difference, RNAi‐*NtSGT1* plants supported population growth of both AAC00‐1 and M6 in comparison to wild‐type *N. tabacum* plants (Fig. 4F and G). These results support that the HR response observed in *N. tabacum* may be caused by the recognition of one or more effectors produced by *A. citrulli* expressing unknown *R* gene(s) that require the function of *NtSGT1*.

## Discussion

Despite the significant economic threat that BFB poses to cucurbit crop production worldwide, limited information is available about the mechanisms of pathogenicity of the causal agent, *A. citrulli *(Bahar and Burdman, 2010; Burdman and Walcott, 2012). An accurate understanding of the molecular host‐pathogen interactions involved in pathogenicity would greatly enhance efforts to manage this disease, particulary as it pertains to durable‐host‐resistance development. This report demonstrates that *N. benthamiana* can function as a surrogate host for studying *A. citrulli* pathogenicity and virulence, while *N. tabacum* may contain non‐host *R* genes that can recognize *A. citrulli* T3S effectors to trigger disease resistance (Fig. 1). By employing *Agrobacterium*‐mediated transient assays, bacterial mutagenesis and growth curve assays on *N.* *benthamiana* plants, we identified Aave_1548 as the first *A. citrulli* T3S effector that contributes significantly to the virulence of *A. citrulli *(Fig. 3).

*N. benthamiana* is an excellent model plant species for studying molecular plant‐microbe interactions. In this study, we demonstrated that the symptoms caused by *A. citrulli* on *N. benthamiana* leaves are similar to those observed on its natural hosts. Spray‐inoculation with cell suspensions containing ~10^8^ CFU/mL of *A. citrulli* onto *N. benthamiana* leaves resulted in necrotic lesions similar to those reported for cucurbit plants (Fig. 1C and E) (Hopkins and Thompson, 2002). By 9 dpi, brown lesions were observed on petioles and stems of inoculated tobacco leaves, as a possible result of systemic infection of *A. citrulli*. The ability of *A. citrulli* to infect and spread via vascular tissue was previously reported in melon seedlings (Bahar *et al.*, 2009b) and squash leaves (Makizumi *et al.*, 2011).

T3S effectors usually make additive contributions to bacterial virulence on host plants. Hence, the deletion of individual T3S effectors may not result in an detectable phenotype on highly susceptible host plants. This is likely due to functional redundancy amongst effectors, or lack of sensitivity of virulence assays (Alfano and Collmer, 2004). Utilization of a weakly virulent pathogen or a less susceptible plant species may substantially aid the detection of virulence contributions of individual T3S effectors. In this study, the *A. citrulli* T3S effector, Aave_1548, was identified as a significant virulence effector when inoculated on *N. benthamiana* leaves. While differences in virulence between strain M6 and M6‐Δ*Aave_1548* could also be detected on melon, inoculation of watermelon leaves with AAC00‐1 wild‐type and mutant strains did not reveal significant contributions by *Aave_1548* nor *Aave_2166* (data not shown) to virulence. Therefore, our results suggest that *N. benthamiana* can be a valuable tool for studying the role of T3S effectors in *A. citrulli *virulence.

We recently showed that all tested Group I strains of *A. citrulli*, including M6, lack functional effector genes *Aave_2166* and *Aave_2708* (Eckshtain‐Levi *et al.*, 2014)*. *Group I strains were also shown to possess a copy of *Aave_3062* that is truncated in the centre of the open reading frame (ORF) (Eckshtain‐Levi *et al.*, 2014). In our study, AAC00‐1‐Δ*Aave_2166* reduced bacterial growth in *N. benthamiana* leaves at 3 dpi, but enhanced population growth at 6 dpi. Some T3S effectors can suppress the activity of other T3S effector proteins when co‐expressed in plant cells (Guo *et al.*, 2009). It is possible that functional Aave_2166 and/or Aave_3062 have an inhibitory effect on other T3S effectors, such as Aave_1548, which could mask their phenotype in Group II strains in cucurbits other than watermelon. Nevertheless, transient co‐expression of Aave_2166‐AAC00‐1, Aave_1548‐AAC00‐1 and Aave_1548‐M6 in pairs failed to trigger different phenotypes compared to individual expression of each effector (data not shown). Further investigation is needed to determine the role *Aave_2166* plays in AAC00‐1 virulence relative to M6 on *N. benthamiana* and whether this effector is involved in the observed restriction of cucurbit host range in Group II strains.

*R*‐gene‐mediated resistance can be an indication of the loss of avirulent T3S effector genes (Kearney *et al.*, 1988; Vera Cruz *et al.*, 2000; Yang *et al.*, 2005). It is also possible that *N. benthamiana* and some cucurbit species have evolved weak *R* genes that can partially recognize Aave_2166, Aave_2708 and Aave_3062 effectors produced by Group II strains. The fact that Group I strains do not express these effectors in a functional state could partially explain the broad cucurbit host range of these strains relative to Group II. In future studies, it would be interesting to express these T3S effector genes in M6 to determine if they reduce virulence on *N. benthamiana* and cucurbits other than watermelon.

*Aave_2166* is a homologue of *avrBsT*, a gene that encodes proteins with acetyltransferase enzyme activity, that was originally cloned from *Xanthomonas campestris *pv.* vesicatoria *(*Xcv*) (Ciesiolka *et al.*, 1999)*. *In *Xcv*, AvrBsT is a critical virulence effector that may target important Arabidopsis or pepper immune signalling components (Cheong *et al.*, 2014; Kim *et al.*, 2010, 2014, 2010, 2014; Szczesny *et al.*, 2010; Thieme *et al.*, 2005). In this study, we demonstrated that *A. citrulli* *Aave_2166* triggers non‐host HR in *N. tabacum*; however, deletion of *Aave_2166* did not significantly compromise the virulence of *A. citrulli* in melon seedling transmission assays (Table 1). Interestingly, an additional T3S effector gene from *A. citrulli*, *Aave_2708*, also encodes a protein belonging to the acetyltransferase family of effectors that is only present in Group II strains of *A. citrulli* (Eckshtain‐Levi *et al.*, 2014). This raises the possibility that *Aave_2166* and *Aave_2708* may have redundant functions when translocated into plant cells. It will be interesting to test if double knockout mutants of *Aave_2166* and *Aave_2708* significantly increase AAC00‐1 virulence on cucurbit hosts other than watermelon. A recent report suggests that the *Xcv* effector, AvrBsT interacts with pepper SGT1, and silencing of pepper SGT1 suppressed AvrBsT‐triggered cell death (Kim *et al.*, 2014). In the current study, *N. tabaccum* SGT1 was also required for Aave_2166‐triggered cell death (Fig. 4B). It remains to be seen whether NtSGT1 also directly interacts with Aave_2166.

Aave_1548 is a homologue of HopW1‐1, originally identified in *Pseudomonas* *syringae* pv. *maculicola* strain ES4326 (Guttman *et al.*, 2002). HopW1‐1 was shown to induce defence responses and resistance in Arabidopsis ecotype Ws, with increased SA accumulation (Lee *et al.*, 2008). More recently, Kang and colleagues showed that HopW1‐1 promotes virulence in Arabidopsis Col‐0 ecoype by disrupting the actin cytoskeleton, which is associated with inhibition of endocytosis and trafficking of specific proteins to vacuoles (Kang *et al.*, 2014). In the current study, *Aave_1548 *significantly contributed to *A. citrulli* virulence in *N. benthamiana* and in melon plants (Fig. 3A–C and Table 1). Future studies should determine whether *A. citrulli* Aave_1548 promotes virulence through interference with the actin cytoskeleton in *N. benthamiana* and cucurbit hosts. Interestingly, the M6 Aave_1548 triggerred a stronger HR in *N. tabacum* than AAC00‐1 Aave_1548. Amino acid sequence alignment between Aave_1548 sequences of Groups I and II strains revealed a small central domain with high polymorphism (14 amino acid differences in a 45‐amino acid region; Fig. S2) (Eckshtain‐Levi *et al.*, 2014). We are currently performing site directed mutagenesis to idenitify the critical amino acid residues that are responsible for differences in HR observed between the two *Aave_1548* homologue.

*N. tabacum *is naturally resistant to *A. citrulli *(Schaad *et al.*, 1978). There are several explanations for the non‐host resistance of *N. tabacum* to *A. citrulli*: (i) the structure of *N. tabacum* leaves could render a physical barrier for the establishment of *A. citrulli *populations; (ii) recognition of conserved pathogen associated molecular patterns (PAMPs) by tobacco pattern recognition receptors (PRRs) and subsequent initiation of PAMP‐triggered immunity (PTI); and/or (iii) the presence of *R* genes that may recognize one or more effectors and initiate ETI. Our data showed that at least three *A. citrulli* T3S effectors triggered HR in *N. tabacum*. Moreover, silencing of *NtSGT1*, a putative common R‐protein chaperone, compromised the resistance of *N. tabacum* to *A. citrulli *strain AAC00‐1 (Fig. 4C). Therefore, ETI contributes, at least partially, to the non‐host resistance of *N. tabacum* to *A. citrulli*. Interestingly, the Group II strain AAC00‐1, but not Group I strain M6, induced weak lesions on inoculated leaves of the *NtSGT1*‐RNAi plant at 9 dpi (Fig. 4D and E). Comparing the whole genome sequences of AAC00‐1 and M6 may help identify other virulence factors that constribute to the host ranges of Groups I and II strains.

In this study, deletion of *Aave_2166* and *Aave_1548* from *A. citrulli* did not completely abolish the non‐host resistance phenotype of *N. tabacum*. Deletion analysis may have been ineffective because multiple *A. citrulli* T3S effectors could be recognized by as yet unidentified *N. tabacum* *R* gene(s). Importantly, isolation and transfer of non‐host resistance genes from *N. tabacum* to cucurbits could potentially offer a novel strategy for controlling BFB. Future studies should focus on identifying other T3S effector genes in *A. citrulli* and to generating *A. citrulli* mutants with multiple deletions of T3S effector genes to determine whether some combinations of impaired effector genes abolish non‐host resistance in *N. tabacum*. Such information could help focus screens for non‐host resistance genes against *A. citrulli*.

In summary, our data support that *Nicotiana* species can be used as surrogate hosts for studying *A. citrulli* pathogenicity. *Agrobacterium*‐mediated transient assays of individual *A. citrulli* T3S effectors on *N. benthamiana* and *N. tabacum* can allow us to characterize the biological functions of T3S effectors and may facilitate the development of new strategies for BFB management.

## Experimental Procedures

### Bacterial strains, plasmids, primers and plant material

Bacterial strains and plasmids used in this study are listed in Table 2. *N. benthamiana* PI 555478 and *N. tabacum* cv. Samsun‐NN plants were propagated from seed in a growth chamber programmed for 16 h light (140 µmol/m^2^/s cool white fluorescent irradiance) at 28 °C and 8 h dark at 25 °C. Hybrid cantaloupe/muskmelon seeds (cv. Athena) were inoculated with *A. citrulli* AAC00‐1 and derived mutants, while melon cv. Ofir were inoculated with *A. citrulli* M6 and derived mutants. Watermelon cv. Sugar Baby was inoculated with all *A. citrulli* strains. Primers used for amplification of ORFs of T3S effector genes from strains AAC00‐1 and M6 are listed in Supplementary Table S1. Other primers used in this study are listed in Supplementary Table S2.

**Table 2:** List of bacterial strains and plasmids.

Strain/Plasmid	Characteristics			References
*Acidovorax citrulli* strains*				
AAC00‐1			Amp^r^, Rif^r^, wild type group II strain	Walcott *et al.* (2000)
M6			Amp^r^, Rif^r^, wild type group I strain	Burdman *et al.* (2005)
AAC00‐1‐Δ*hrcC*			Amp^r^, Rif^r^, Km^r^, AAC00‐1 mutant defective in *hrcC*	Johnson *et al.* (2011)
M6‐Δ*hrcV*			Amp^r^, Rif^r^, Km^r^, M6 mutant defective in *hrcV*	Bahar and Burdman (2010)
AAC001*‐*Δ*Aave_2166*			Amp^r^, Rif^r^, Km^r^, AAC00‐1 mutant defective in *Aave_2166*	This work
AAC00‐1‐Δ*Aave_1548*			Amp^r^, Rif^r^, Km^r^, AAC00‐1 mutant defective in *Aave_1548*	This work
AAC00‐1‐ Δ*Aave_1548* (Pvsp61‐Aave_1548)			Amp^r^, Rif^r^, Km^r^, Gm^r^, AAC00‐1 mutant defective in *Aave_1548 *that was complemented with pVSP61*‐GM‐Aave_1548*	This work
M6‐Δ*Aave_1548*			Amp^r^, Rif^r^, Km^r^, M6 mutant defective in *Aave_1548*	This work
*Escherichia coli* strains†				
DH5α		F^‐^ endA1 glnV44 thi‐1 recA1 relA1 gyrA96 deoR nupG Φ80d*lacZ*ΔM15 Δ(*lacZYA‐argF*)U169, hsdR17(r_K_^‐^ m_K_^+^), λ–		Invitrogen Inc.
DH5α (RK600)		Cm^r ^, helper plasmid		Figurski and Helinski (1979)
*Agrobacterium tumefaciens* strains‡				
GV2260		C58 background, Rif^r^		This study
LBA4404		TiAch5		Clontech Inc.
*Pseudomonas syringae tabaci* strain§				
*P. syringe *pv*. tabaci* (*Pta 11528*)		Rif^r^, wild type		Wei *et al.* (2007)
Plasmids				
pEG101/SacB/R		Km^r^, toxic on high concentration sucrose, expression vector		Traore and Zhao (2011)
pLVC18L‐Des		Suicide vector, Tet^r^, low copy		Zhao *et al.* (2011)
pVSP61‐Des‐GM		Km^r^, Broad range expression vector		Century *et al.* (1995)
pORE E3		Km^r^, plant expression vector,		Coutu *et al.* (2007)
pORE‐E2‐SGT1‐RNAi		Km^r^, RNAi construct for silencing *NtSGT1*		This work
pLVC18L‐Aave_1548‐Kan		Km^r^,		This work
pLVC18L‐Aave_2166‐Kan		Km^r^,		This work
pVSP61‐GM‐Aave_1548		Km^r^, Gm^r^		This work

Km^r^: kanamycin resistant, Gm^r^: gentamycin resistant, Rif^r^: rifampicin resistant, Tet^r^: tetracycline resistant, Cm^r^: chloramphenicol resistant.

* All *A. citrulli* strains were grown on NA at 28 °C.

† *E. coli* strains were grown on LB plates at 37 °C.

‡ *A. tumefaciens* strains were grown on LB plates at 28 °C.

§ *P. syringae *pv.* tabaci* strains were grown on NYGA medium at 28 °C.

### *Agrobacterium*‐mediated transient assays in *Nicotiana* plants

The ORFs of T3S effector genes were PCR amplified from AAC00‐1 and M6 genomic DNA. The amplified DNA fragments were cloned into the Topo ENTR/D vector (Invitrogen, Carlsbad, CA) according to manufacturer’s instructions. The sequences of all cloned genes, including the construct described below, were verified by DNA sequencing at the core facility of the Virginia Bioinformatics Institute (Blacksburg, VA). The effector genes were sub‐cloned into the plant expression vector pEG101‐SacB/R (Traore and Zhao, 2011) by LR reaction (Invitrogen). The pEG101‐SacB/R vector has a C‐terminal YFP fusion. After subcloning, the effector genes were fused to the N‐terminus of the YFP gene as described (Traore and Zhao, 2011). Recombinant plasmids were electroporated into *Agrobacterium tumefaciens* strain GV2260 as described previously (Traore and Zhao, 2011). Transient expression assays in *N. benthamiana* or *N. tabacum* plants were performed as described previously (Traore and Zhao, 2011). Briefly, the *A. tumefaciens* strains were streaked onto Luria‐Bertani (LB) agar supplemented with appropriate antibiotics and incubated at 28 °C for 2 days. Bacterial cells were harvested and resuspended in induction buffer (10 mM MgCl_2_, 10 mM MES [pH 5.6] and 100 µM acetosyringone) and incubated for 3 h at room temperature. Bacterial cell suspensions were then adjusted to OD_600 _= 0.6 (~6 × 10^8^ CFU/mL) and infiltrated through stomata into intercellular spaces of fully expanded *N. benthamiana* leaves using a 1 mL, blunt‐end syringe. The inoculated plants were incubated at 25 °C under continuous light for 20 h–48 h before observing expressed proteins or cell death phenotypes. At least three plant leaves have been inoculated in each experiment. The experiment has been repeated three times. The fluorescent signal of the effector‐YFP fusion protein was observed 20 h after inoculation by fluorescence microscopy (Zeiss Axio Observer.A1, Carl Zeiss MicroImaging, Inc., Thornwood, NY).

### Western blot analysis

The effector genes cloned in pEG101‐SacB/R could express the effecor‐YFP‐HA fusion proteins, which can be detected with anti‐HA antibodies by Western blot. At 20 h after *Agrobacterium* infiltration, the transiently expressed effecor fusion proteins were extracted by grinding three leaf discs (19 cm diameter) in 100 µL 3 × Laemmli buffer containing 16% β‐mercaptoethanol. Also, 20 µL protein samples were separated on a 10% SDS‐PAGE gel, and blotted to a PVDF membrane. The blot was probed with anti‐HA‐HRP (Sigma, St. Louis, MO; H6533; 1:500), and the signal was detected with using SuperSignal^® ^West Pico Chemiluminescent Substrate (Thermo Scientific, Waltham, MA). The chemiluminescent signals were exposed to autoradiography film (Genesee Scientific, San Diego, CA) using a Kodak film processor (Kodak, A Walsh Imaging, Inc, Pompton Lakes, NJ). Another 20 µL protein samples were also separated on a 10% SDS‐PAGE gel, and stained with comassie blue to confirm the equal loadings.

### Knockout of T3S effectors by marker‐exchange mutagenesis and mutant strain complementation

To knockout *A. citrulli* T3S effectors, 1.4 kb and 1.5 kb DNA fragments from regions flanking the ORFs of effector genes *Aave_2166* and *Aave_1548*, respectively, were amplified using the following primers: Aave2166_f1/Aave2166_R2 (upstream region), and Aave2166_f5/Aave2166_R6 (downstream region) for gene *Aave2166*; and Aave1548_f1/Aave1548_R2 (upstream region), and Aave1548_f5/Aave1548_R6 (downstream region) for gene *Aave_1548*. A kanamycin (Km) resistance gene (*nptII*) was amplified from pDSK519‐GFP (Matthysse *et al.*, 1996) with primers kan_for and kan_rev. The two flanking fragments from both genes were then fused to the *nptII* gene by overlap PCR (Higuchi, 1990). The derived cassettes were cloned into the PCR8/GW‐Topo vector (Invitrogen), and cloned into the suicide vector pLVC18L‐Des (Staskawicz *et al.*, 1987; Zhao *et al.*, 2011) using LR clonase (Invitrogen). The derived construct was then mobilized into *A. citrulli* strains by tri‐parental mating as previously described (Ditta *et al.*, 1980). Double crossover mutants (Supplementary Fig. S1A) were selected using marker‐exchange mutagenesis as previously reported (Zhao *et al.*, 2011). The mutant genotypes were confirmed by PCR analysis (Supplementary Fig. S1B, C and D). *A. citrulli* strains that contained impaired genes were selected on nutrient agar (NA) medium (Thermo Fisher Scientific Inc, Waltham, MA) supplemented with rifampicin (100 µg/mL) and kanamycin (50 µg/mL). To complement the knockout mutants of Aave_1548, the *Aave_1548* effector gene, including its native promoter and full ORF, was amplified by PCR using primers Aave_1548comp for and Aave_1548comp rev. The PCR products were cloned into the PCR8/GW‐Topo vector (Invitrogen), and then sub‐cloned into the broad host range vector pVSP61‐Des‐GM (Century *et al.*, 1995). Successful transformants were selected on NA supplemented with gentamycin at 50 µg/mL. The derived constructs were then mobilized into *A. citrulli* by conjugation. An M6 mutant impaired in *Aave_1548* was generated as previously described (Bahar *et al.*, 2009b). An internal fragment of *Aave_1548* (which does not span the 3′ and 5′ ends of this gene) was PCR amplified with primers Inter1548F and Inter1548R. The PCR product was cloned into pTZ57R/T (Thermo Fisher Scientific Inc), verified by sequencing, excised with appropriate restriction enzymes, and then cloned into pJP5603 (Penfold and Pemberton, 1992) conferring kanamycin resistance. Transformation into strain M6 was carried out as described previously (Bahar *et al.*, 2009b). Putative mutants were selected on NA with kanamycin and verified by Southern blot analysis.

### Virulence assays on *N. benthamiana*, *N. tabacum *and watermelon plants

*A. citrulli* strains AAC00‐1, M6, the derived mutants and complemented strains, as well as *Pseudomonas syringae *pv.* tabaci *(*Pta *11528) were grown on NA supplemented with rifampicin (100 µg/mL) at 28 °C for 48 h. For inoculation, bacterial cells were harvested from the plates and suspended in 10 mM MgCl_2_. The inoculum concentrations were adjusted to OD_600 _= 0.3 (~0.3 × 10^8^ CFU/mL) for the HR assay and (~0.2 × 10^5^ CFU/mL) for the infiltration‐based virulence assay. For spray‐inoculation, bacterial concentrations were adjusted to OD_600_ = 0.2 (~2 × 10^7^ CFU/mL) and 0.01% of Silwet L‐77 (LEHLE SEEDS, Round Rock, TX, USA) was added to the cell suspensions. Before inoculation, 4‐week‐old *N. benthamiana*, *N. tabacum*, and 3‐week‐old watermelon plants were covered with plastic bags for 12 h to maintain high relative humidity. At least three plants have been used for each inoculation in all experiments. The plants were then spray‐inoculated using a atomizing minibottle and covered with a plastic bag for 24 h. Inoculated plants were maintained in a growth chamber as described above. Two hours after inoculation, spray‐inoculated leaves were harvested, soaked in 5% hydrogen peroxide solution for 2 min, then washed in distilled water at least three times before leaf discs (0.33 cm^2^) were collected. Bacteria were extracted from nine leaf discs collected from three plants ground in the tube and populations were determined by ten‐fold serial dilution of cell suspensions followed by spread plating 100 µL aliquots onto NA media amended with rifampicin. Bacterial populations were also determined 3 days and 6 days post‐inoculation using the same procedure.

### Melon seed transmission assays

Melon cv. Ofir and hybrid cantaloupe/muskmelon (cv. Athena) seeds were used for inoculation of M6‐ and AAC00‐1‐derived strains, respectively. Seeds were incubated for 2 h with gentle agitation in 50 mL tubes containing 10 mL suspensions of *A. citrulli* (~1 x 10^6^ and ~1 × 10^7 ^CFU/mL for AAC00‐1‐derived and M6‐derived strains, respectively). As a control, melon seeds were incubated in sterile distilled water for 2 h. Following incubation, seeds were air‐dried in a laminar flow for 4 h. Seeds were sown in 600 mL pots filled with peat (one seed per pot). The pots were kept in a greenhouse at 26 °C–28 °C for 12 days and then the above ground parts of the seedlings were collected and their fresh weight was determined. We have previously shown that seedling fresh weight directly correlates with disease severity in *A. citrulli* seed transmission assays (Bahar *et al.*, 2009b). Disease severity was quantified using a 0 to 7 scale, based on the fresh weight of inoculated plants relative to the average weight of non‐inoculated control plants (Bahar *et al.*, 2009b) where: 0 = weight higher than 90% of average control weight; 1 to 5 = weight equal to 76%–90%, 61%–75%, 46%–60%, 31%–45% and 16%–30% of average control weight, respectively; 6 = weight equal to or lower than 15% of average control weight; 7 = dead seedling.

### Developing an RNAi construct for silencing the *NtSGT1* gene in *N. tabacum*

A partial *NtSGT1* gene fragment was amplified by PCR from the cDNA of *N. tabacum* (cv. Samsun‐NN) using primers NtSgt1‐H3‐RI and NtSgt1‐Xba‐Sal. The PCR product was digested with *Xba*I and *Hind*III to obtain the anti‐sense strand DNA or *Eco*RI and *Sal*I to obtain the sense DNA fragment, and sub‐cloned into the corresponding restriction sites of Topo‐Cannibal vector as described previously (Xu *et al.*, 2011). The derived Topo‐Entry construct consisted of a DNA fragment carrying an anti‐sense‐*NtSGT1 *DNA fragment, a castor bean intron spacer sequence, and a sense‐*NtSGT1* DNA fragment. The DNA fragment was released from the Topo‐Cannibal vector using *Not*I, and sub‐cloned into plasmid pORE‐E2 (Coutu *et al.*, 2007) after digestion with *Sal*I and filled‐in with the Klenow enzyme. The derived construct was named pORE‐E2‐SGT1‐RNAi.

### Generating *N. tabacum‐*RNAi*‐NtSGT1 *transgenic plants

The pORE‐E2‐SGT1‐RNAi construct was transferred into *A. tumefaciens* strain LBA4404 by electroporation (Traore and Zhao, 2011). *Agrobacterium*‐mediated leaf disc transformation was performed as described previously (Horsch *et al.*, 1989). Briefly, fully expanded leaves of 4‐week‐old *N. tabacum *plants (cv. Samsum‐NN) were collected and surface sterilized with 10% sodium hypochlorite. Disinfected leaves were cut with a sterilized razor blade into small leaf discs (about 1 cm^2^) that were inoculated by incubation in an *Agrobacterium* suspension (OD_600 _= 0.1, ~1 × 10^8^ CFU/mL). Inoculated leaf discs were incubated on MS medium (PhytoTechnology Laboratories, Shawnee Mission, KS, USA) in the dark at 25 °C for 2 days–3 days. After co‐cultivation, the transformed leaf discs were washed with liquid MS medium and cultured on MS medium supplemented with kanamycin (300 µg/mL), carbenicillin (500 µg/mL), 1‐naphthaleneacetic acid (100 mM), and 6‐benzylaminopurine (50 mM) for 4 weeks. Regenerated transgenic shoots were further selected on MS medium supplemented with kanamycin (100 µg/mL) and carbenicillin (300 µg/mL). Fully rooted transgenic plants were transplanted into soil and maintained in a growth chamber under conditions of 16 h of light daily at 28 °C, and 8 h dark at 25 °C as described above.

### Monitoring the silencing of *NtSGT1* by Reverse Transcriptase‐Polymerase Chain Reaction (RT‐PCR)

Total mRNAs of the pORE‐E2‐SGT1‐RNAi transgenic, and non‐transgenic control plants were extracted using the TRIzol reagent (Invitrogen) according to manufacturer’s instructions. The total RNA samples were treated with DNase I (Zymo Research, Irvine, CA, USA). First‐strand cDNA synthesis was performed using the DyNAmo cDNA Synthesis Kit (Thermo Scientific Inc., Pittsburgh, PA, USA). *NtSGT1* transcripts were detected by qPCR with primers NtSGT1‐Cter For and NtSGT1‐Cter Rev, which amplify an *NtSGT1* fragment outside the region used for developing the RNAi construct. The *N. tabacum *actin gene served as a reference and was amplified using primers Nt_actin For and Nt_actin Rev. PCR was performed using one cycle at 98 °C (3 min), followed by 25 cycles at 98 °C (1 min), 55 °C (1 min), and 72 °C (1 min), and by one cycle at 72 °C (7 min). Similar PCR conditions were also used for checking the mutants of *A. citrulli* strains (Supplementary Fig. S1). The PCR products were separated on 0.8% agrose gel, stained with ethodium brominde, and visualized using the Gel‐Document Image System (Bio‐Rad, Hercules, CA, USA).

### Statistical analysis

To determine if there were significant differences in virulence between bacterial strains in *in planta* assays, treatment means were analysed by the Tukey–Kramer HSD multiple comparison procedure using the JMP software (Version 7, SAS Inst., Cary, NC, USA).

## Supporting information


**Fig. S1** Diagram of marker exchange mutagenesis of T3S effectors of strain AAC00 1 and PCR validation. (A) The diagram of the contructs used for marker exhange mutagenesis. The primer binding sites are indicated as arrows. The sequence of Polymerase Chain Reaction (PCR) primers is listed in Table S2. (B and C) The *Aave_1548* and *Aave_2166* mutants were genotyped using primers flanking the effector gene (B, *Aave_1548*; C, *Aave_2166*). The *nptII* gene fragment that was used to replace the open reading frames (ORFs) of the effector genes is larger than the replaced ORFs thus, amplification with flanking regions of the mutated effectors give bigger bands than those amplified from the wild type strain. (D) The presence of the *nptII* gene was confirmed in the mutants using *nptII* (kan) specific primers (Supplementary Table S2).

**Fig. S2** Amino acid sequence alignment of Aave_1548 effectors from *A. citrulli* AAC00 1 and M6. A small domain that is highly polymorphic between the two Aave_1548 homologues is highlighted in yellow.

**Table S1** List of the putative AAC00 1 T3S effector genes, their annotation, and primers used to amplify the open reading frames (ORFs) from AAC00 1 and M6.

**Table S2** List of additional primers used in this study.
