# Supplementary figures and images for: Nicotiana species as surrogate host for studying the pathogenicity of Acidovorax citrulli, the causal agent of bacterial fruit blotch of cucurbits
Source: Mol Plant Pathol. 2019 Apr 1;20(6):800–14. doi: 10.1111/mpp.12792 (PMC6637898; doi:10.1111/mpp.12792)

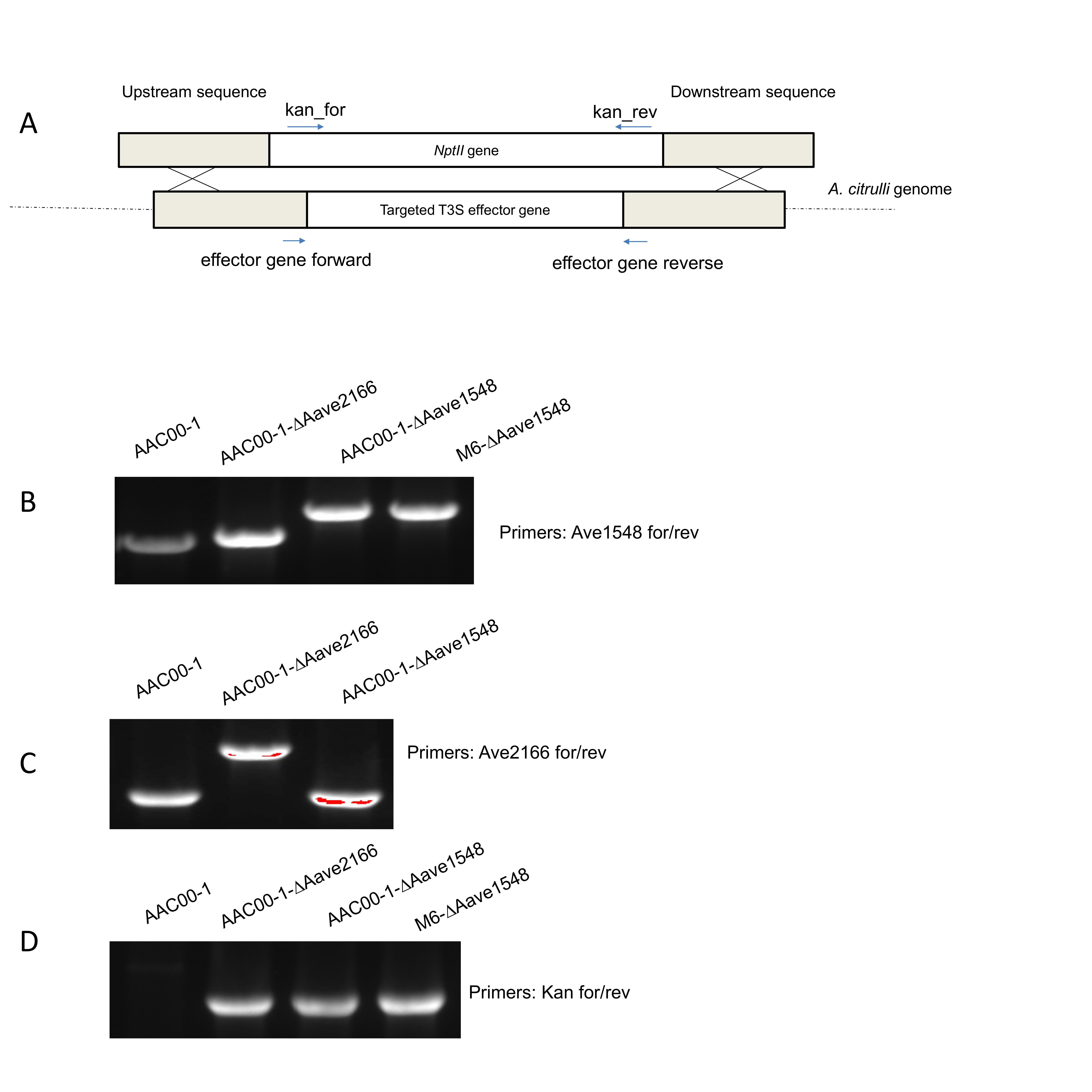

Supplement: Supplementary file 1 — Fig. S1 Diagram of marker exchange mutagenesis of T3S effectors of strain AAC00 1 and PCR validation. (A) The diagram of the contructs used for marker exhange mutagenesis. The primer binding sites are indicated as arrows. The sequence of Polymerase Chain Reaction (PCR) primers is listed in Table S2. (B and C) The Aave_1548 and Aave_2166 mutants were genotyped using primers flanking the effector gene (B, Aave_1548; C, Aave_2166). The nptII gene fragment that was used to replace the open reading frames (ORFs) of the effector genes is larger than the replaced ORFs thus, amplification with flanking regions of the mutated effectors give bigger bands than those amplified from the wild type strain. (D) The presence of the nptII gene was confirmed in the mutants using nptII (kan) specific primers (Supplementary Table S2). [file MPP-20-800-s001.tif]

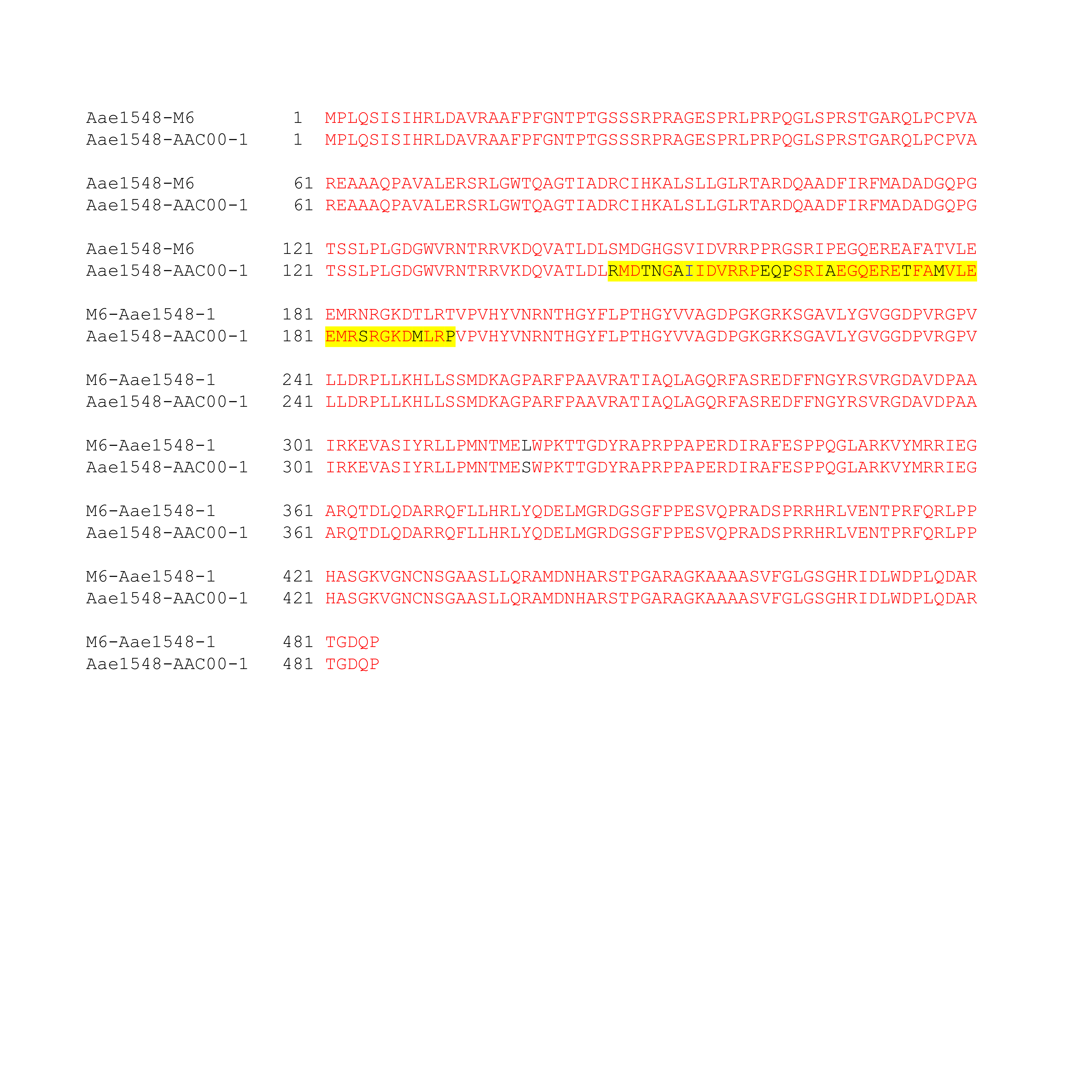

Supplement: Supplementary file 2 — Fig. S2 Amino acid sequence alignment of Aave_1548 effectors from A. citrulli AAC00 1 and M6. A small domain that is highly polymorphic between the two Aave_1548 homologues is highlighted in yellow. [file MPP-20-800-s002.tif]
