# Supplementary material for: Nicotiana species as surrogate host for studying the pathogenicity of Acidovorax citrulli, the causal agent of bacterial fruit blotch of cucurbits
Source: Mol Plant Pathol. 2019 Apr 1;20(6):800–14. doi: 10.1111/mpp.12792 (PMC6637898; doi:10.1111/mpp.12792)
Supplement: Supplementary file 3 — Table S1 List of the putative AAC00 1 T3S effector genes, their annotation, and primers used to amplify the open reading frames (ORFs) from AAC00 1 and M6. [file MPP-20-800-s003.docx]

**Table S1.** List of the putative AAC00-1 T3S effector genes, their annotation, and primers used to amplify the open reading frames from AAC00-1 and M6.

| Locus Tag | **Gene and Description** | **Primer Sequences^a^** |
| --- | --- | --- |
| Aave0277 | Type III effector XopAG (*Xanthomonas citri*) | 5’-cacc**GGATCC**ATGAGAAAAGAACTAGCCAGCTGT-3’  5’-**GTCGAC**GGCAGGCCTGCCCGGCAGCCCGGCCT-3’ |
| Aave1548 | Type III secreted effector HopW1-1 (*Pseudomonas syringae* pv*. maculicola)* | 5’-cacc**TCTAGA**ATGCCTCTACAGTCCATTTCCAT-3’  5’-**GTCGAC**TGGTTGATCCCCCGTCCGAGCAT-3’ |
| Aave2166 | Avirulence protein AvrBsT (*Xanthomonas campestris* pv*. vesicatoria*) | 5’-cacc**AGATCT**ATGAAGAATTTCATGCGATCGAT-3’5’-**GTCGAC**TTCGATAGCTTTTCTGATTTTTCTCA-3’ |
| Aave2173 | Avirulence protein AvrBs1 (*Xanthomonas campestris* pv*. vesicatoria*) | 5’-cacc**GGATCC**ATGCTGGAGAAAAAGAGGCAGGT-3’  5’-**GTCGAC**GGCAACATGCTGCACCTTGGAGGA-3’ |
| Aave2708 | Type III effector XopJ (*Xanthomonas campestris* pv*. vesicatoria*) | 5’-cacc**GGATCC**ATGGGTCTATGCGTTTCAAA-3’  5’-**GTCGAC**TGACTGGCGATCAGAGATAGCT-3’ |
| Aave2876 | Type III effector HopH1 (*Pseudomonas syringae* pv*. tomato*) | 5’-cacc**GGATCC**GTGAACGTCCATCTGCATGCCA-3’  5’-**GTCGAC**GTCGCTTCGGTAACCTGAATAAT-3’ |
| Aave3062 | Putative avirulence protein AvrRxo1 (*Xanthomonas oryzae* pv*. oryzicola*) | 5’-cacc**GGATCC**GCCCGCCTCCTCGACCAGGAT-3’  5’- **GTCGAC**AGTCAGTGAACTATGGGCGGCCA-3’ |
| Aave3237 | Type III effector HopAJ2 (*Pseudomonas syringae* pv*. phaseolicola*) | 5’-cacc**GGATCC**ATGCAACGAAGAAACACGACGA-3’  5’-**GTCGAC**GGGCGCCTGCAGTCGCTGCAGCA-3’ |
| Aave3452 | Avirulence protein AvrPphe (*Xanthomonas axonopodis* pv*. citri*) | 5’-cacc**GGATCC**ATGGCAACTTTCATCATCTCGTCCAT-3’  5’-**GTCGAC**GGACGTTCTCCGGCGGAGCCGGCA-3’ |
| Aave3462 | Type III effector PopP2 protein (*Ralstonia solanacearum*) | 5’-cacc**GGATCC**ATGCCCCGTTCCGTCACATCCT-3’  5’-**GTCGAC**GTGCCGATACCAGTCGCGCCA-3’ |
| Aave4728 | Type III effector XopX (*Xanthomonas* sp.) | 5’-cacc**AGATCT**ATGTCTGCGATCAACAGTTCCT-3’  5’-**CTCGAG**GGGGGCCACGGGCGCGTGGCGCGGAA-3’ |

^a^Restriction enzyme recognition sites included at the ends of each primer are bolded. Extra “cacc” bases were included at all forward primers to facility the cloning into the Topo EntrD vector (Invitrogen).
