# Supplementary material for: Nicotiana species as surrogate host for studying the pathogenicity of Acidovorax citrulli, the causal agent of bacterial fruit blotch of cucurbits
Source: Mol Plant Pathol. 2019 Apr 1;20(6):800–14. doi: 10.1111/mpp.12792 (PMC6637898; doi:10.1111/mpp.12792)
Supplement: Supplementary file 4 — Table S2 List of additional primers used in this study. [file MPP-20-800-s004.docx]

**Table S2.** List of additional primers used in this study

| Primer names | Primer sequences |
| --- | --- |
| Aave2166_f1 | 5’-gaagcccacgtgcgcgtgctggcc-3’ |
| Aave2166_R2 | 5’CCTACACAATCGCTCAAGACGTGCCATTCTCAATTGCAAATATTCAG-3’ |
| Aave2166_f5 | 5’ggaccatggctaattcccatgtcgcaccgggaatgataatccccgg-3’ |
| Aave2166_R6: | 5’- CGCTGTCGTAGCCCACGGTGATGTTCG-3’ |
| Aave1548_f1 | 5’-cccggatcgaaaggcgagcgcacg-3’ |
| Aave1548_R2 | 5’-CCTACACAATCGCTCAAGACGTGTGCTGCAGCGTGCCATGGACAA-3’ |
| Aave1548_f5 | 5’-ggaccatggctaattcccatgtcccttggggacgggggagtctggga-3’ |
| Aave1548_R6 | 5’-GGTGAGCCAGTGGAAGCGCGCCCGG-3’ |
| Aave_1548comp for | 5’-CACCTCTAGACACCAGCGCGTCGAACAGA-3’ |
| Aave_1548comp rev | 5’-GTCGACTGGTTGATCCCCCGTCCGAGCAT-3’ |
| kan_for | 5’-CACGTCTTGAGCGATTGTGTAGG-3’ |
| kan_rev | 5’-GACATGGGAATTAGCCATGGTCC-3’ |
| Inter1548F | 5’-GCGTCGACGAAGATTTCTTCAACGGCTA-3’ |
| Inter1548R | 5’-CCGGTACCCATCAACTCGTCCTGGTAGA-3’ |
| NtSgt1-H3-RI | 5’-AGCTACCaagcttGAATTCGCTGGAGAACTACCTAATCAGTC-3’ |
| NtSgt1-Xba-Sal | 5’GATGCTCTAGAGTCGACTTCGAGAGATGTCCAGTGTAAGG-3’ |
| NtSGT1-Cter For | 5’-AGAGAGTCTGCTGTAGTGCAGAG-3’ |
| NtSGT1-Cter Rev | 5’-TTCTTCAGCTCCATGCCATCTGGA-3’ |
| Nt_actin For | 5’-TTGACGGAAAGAGGTTAT-3’ |
| Nt_actin Rev | 5’-GTTGGAAGGTGCTGAGAG-3’ |
